# Implantation of ventricular assist device for systemic right ventricular failure in a patient with transposition of the great arteries and post-Mustard procedure: a case report

**DOI:** 10.1186/s40981-018-0194-x

**Published:** 2018-07-25

**Authors:** Kazutomo Saito, Hiroaki Toyama, Nozomu Abe, Azusa Sunouchi, Yutaka Ejima, Masanori Yamauchi

**Affiliations:** 1 Anesthesiology and Perioperative Medicine, Tohoku University School of Medicine, 2-1 Seiryo-cho, Aoba-ku, Sendai, Miyagi 980-8575 Japan; 2 Department of Anesthesiology, Tohoku University Hospital, 1-1 Seiryo-cho, Aoba-ku, Sendai, 980-8574 Japan; 3 Division of Surgical Center and Supply, Sterilization, Tohoku University Hospital, 1-1 Seiryo-cho, Aoba-ku, Sendai, 980-8574 Japan

**Keywords:** Transposition of the great arteries, Ventricular assist device, Mustard procedure, Systemic right ventricular failure

## Abstract

**Background:**

Ventricular assist device (VAD) is usually attached by an inflow cannula to the apex of the systemic left ventricle (LV), but very few cases with implantation of the VAD in the morphologic right ventricle (RV) have been described.

**Case presentation:**

We describe the case of a 41-year-old male who developed severe systemic RV failure related to a Mustard procedure he had as an infant for treatment of TGA. His heart failure was refractory and irreversible, and therefore, he underwent VAD implantation for systemic RV support. Although the patient developed pulmonary congestion on postoperative day (POD) 5, he was discharged on POD 60. He is now looking forward to receiving heart transplantation.

**Conclusions:**

Placement of a VAD for systemic RV failure could be a life-saving treatment in adult patients with heart failure due to congenital heart disease.

## Background

After an atrial switch operation (Mustard or Senning operation) for correction of transposition of the great arteries (TGA) or in patients with congenitally corrected TGA (CCTGA), discordant atrioventricular and ventriculoarterial connections are both present. Owing to this double discordance, a reconnection of the heart vessels is necessary to allow for proper blood circulation. This can be done with the Mustard or Senning procedure, which will help prevent cyanosis. Patients who undergo such arterial switch procedures are surviving to adulthood, but they often develop RV heart failure a few decades later. Implantation of ventricular assist device (VAD) is a useful therapy for patients with end-stage heart failure as a bridge to heart transplantation. However, adult congenital heart disease (ACHD) patients with TGA post-Mustard procedure or CCTGA have unique anatomical and physiological characteristics that can make clinical management or surgical procedures more challenging.

## Case presentation

**Fig. 1:**
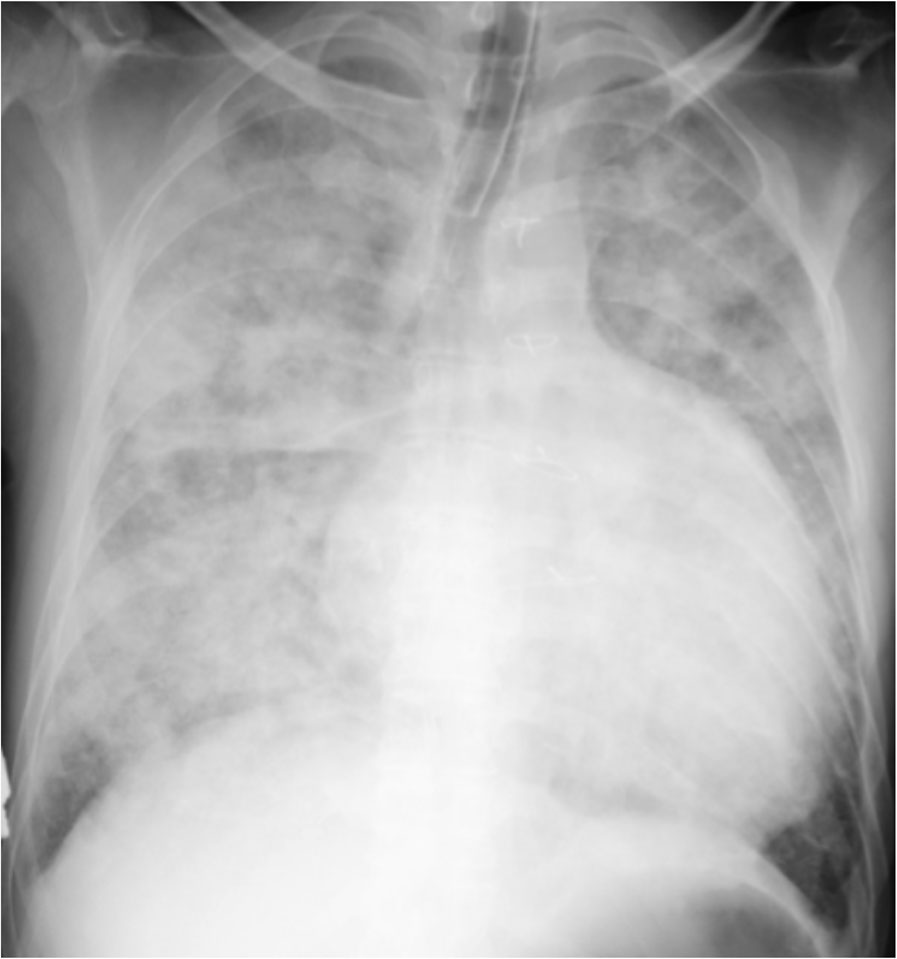
Chest radiography shows pulmonary edema and an extremely dilated ventricular shadow at the onset of severe right ventricular failure

The patient was a 41-year-old man (height 174.2 cm, body weight 60 kg) with dextro-TGA who underwent balloon atrioseptostomy treatment at birth because of desaturation. He also had a Mustard procedure (interatrial baffle) at 10 months of age and required surgical repair for pulmonary vein stenosis at 2 years of age. At 41 years of age, he developed severe systemic RV failure and pulmonary edema (Fig. 1). He was treated with inotropic agents, mechanical ventilation, intra-aortic balloon pumping, and continuous hemodiafiltration. His hemodynamic status was stabilized with these treatments, but he remained inotrope-dependent, and his condition gradually worsened despite optimal medical therapy. After a medical team preoperative meeting, he was scheduled for a VAD implantation of the RV as a bridge to orthotropic cardiac transplantation.

**Fig. 2:**
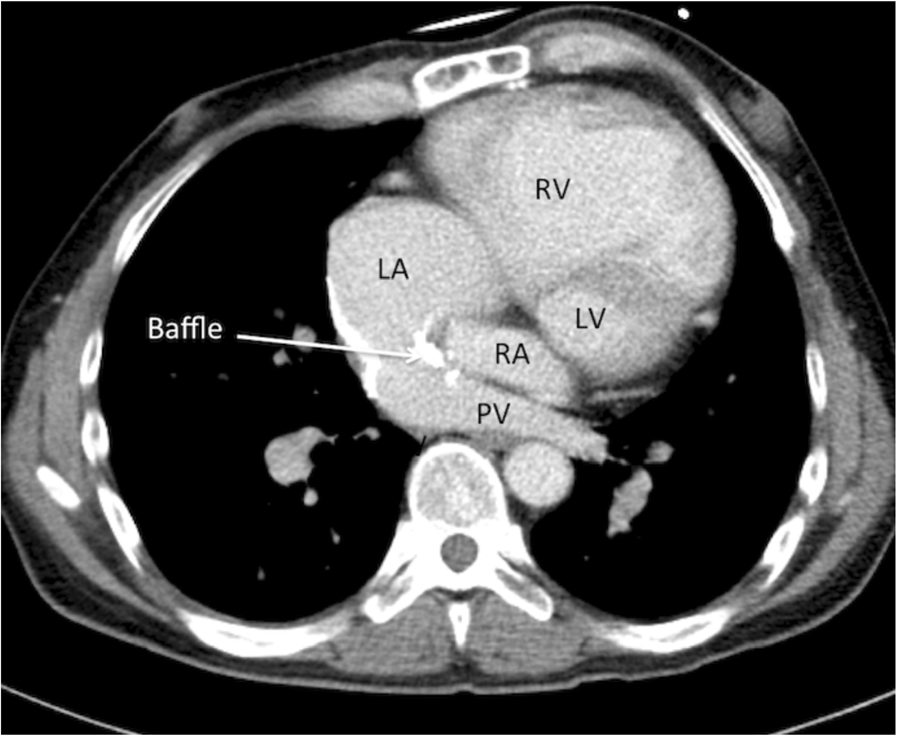
Computed tomography just before systemic ventricular assist device implantation shows baffle calcification, a slightly narrowed pulmonary vein, and dilated left atrium and right ventricle. *LA* left atrium, *RV* right ventricle, *RA* right atrium, *LV* left ventricle, *PV* pulmonary vein

Preoperative cardiac catheterization indicated elevated pulmonary arterial wedge pressure and severely impaired systemic RV function with infusion of dobutamine (3 μg/kg/min) and carperitide (24 ng/kg/min), a recombinant atrial natriuretic peptide with vasodilating and diuretic activity (Table 1). Echocardiography identified the dilated RV, preserved pulmonary ventricle (morphological left ventricle), and increased baffle flow velocity (Table 1). Chest radiography and computed tomography scan showed a severely dilated RV (Figs. 1 and 2).

**Table 1:** Perioperative echocardiographic and cardiac catheterization parameters

	Before surgery	Postoperative day 90
Echocardiography		
Right ventricle		
End-diastolic diameter (mm)	61	61
Ejection fraction (%)	19	−
Fractional area change (%)	10	1
TAPSE (mm)	12.1	6.9
LAD (mm)	54	30
Left ventricle		
End-diastolic diameter (mm)	49	35
Ejection fraction (%)	72	67
Fractional area change (%)	40.1	48
IVC (mm)	21	15
Baffle flow (m/s)	1.52	0.9
Others	Moderate AR, mild TR	−
Catheterization		
Right atrial pressure (mmHg)	7	10
Left ventricular pressure (mmHg)	46/8	46/14
PAP (s/d/m) (mmHg)	48/24/32	46/22/32
PAWP (mmHg)	23	18
Arterial pressure (s/d/m) (mmHg)	100/68/83	87/73/81
$S\overline{v}{O}_2$ (%)	64.6	66.8
Cardiac output (L/min)	4.6	4.9
Cardiac index (L/min/m^2^)	2.8	2.9
SVR (WU)	16.5	14.5
PVR (WU)	2.0	2.9
Circulatory support	Dobutamine 3 μg/kg/min	Jarvik 2000 (VAD)

*Abbreviations*: *TAPSE* tricuspid annular plane systolic excursion, *LAD* left atrial end-systolic diameter, *IVC* inferior vena cava diameter, *AR* aortic valve regurgitation, *TR* tricuspid valve regurgitation, *PAP* pulmonary artery pressure, *s/d/m* systolic/diastolic/mean, *PAWP* pulmonary artery wedge pressure, $S\overline{v}{O}_2$ mixed venous oxygen saturation, *SVR* systemic vascular resistance, *WU* Wood unit, *PVR* pulmonary vascular resistance

In the operating room, a continuous monitoring of electrocardiography, SpO_2_, systemic arterial pressure via a right radial artery catheter, bispectral index (BIS; Medtronic, Minneapolis, MN), and regional cerebral oxygen saturation at the right and left forehead (INVOS™ 5100C, Somanetics, USA) was initiated before anesthesia induction. After administration of 100% oxygen, general anesthesia was induced slowly by intravenous administration of 3 mg of midazolam, 0.5 mg of fentanyl, and 50 mg of rocuronium. Additionally, dobutamine (3.3 μg/kg/min), nicorandil (1.1 μg/kg/min), which has the dual properties of a nitrate and a potassium channel opener, and carperitide (33 ng/kg/min) were continuously administered to minimize anesthetic-induced cardiac depression. During anesthesia induction and tracheal intubation, his circulatory and respiratory status remained stable. After tracheal intubation, a transesophageal echocardiography probe was inserted. Then, a central venous catheter and right heart catheter were inserted via the right internal jugular vein. The latter catheter (Swan Ganz catheter) was guided to the right pulmonary artery under fluoroscopic control because of his unique anatomy with interatrial baffle.

His anesthesia depth was controlled at a BIS of 40–60 by a continuous infusion of propofol (3–5 mg/kg/h), remifentanil (0.2–0.3 μg/kg/min), and rocuronium (7 μg/kg/min).

A standard median sternotomy was performed. Because his heart was heavily adherent, the cardiac surgeons had difficulty in adhesiolysis. The systemic aorta and systemic atrium were cannulated, and cardiopulmonary bypass (CPB) was instituted. At first, the patient’s aortic valve was replaced with a bioprosthetic valve for moderate aortic valve regurgitation. Subsequently, the trabeculae carneae of the RV, which could disturb the flow to the inflow cannula, were resected via the RV apex; the inflow cannula was positioned at the apex of the RV. The outflow cannula graft was anastomosed to the ascending aorta, and then a Jarvik 2000 VAD (Jarvik Heart, Inc., New York, NY, US) was implanted.

At the weaning of the CPB, infusion of dobutamine (3.3 μg/kg/min) and inhalation of nitric oxide (20 ppm), to decrease afterload of the pulmonary LV, was started. The CPB weaning was not difficult, and the VAD was successfully commenced at a pump speed of 10,000 rpm (3–5 L/min). The aortic cross-clamping, CPB, surgery, and anesthesia lasted 200, 296, 628, and 807 min, respectively. Estimated blood loss and urine output were 2051 and 2390 mL, respectively. Overall, 900 mL of crystalloid, 900 mL of colloid, 960 mL of packed red blood cells, 560 mL of fresh frozen plasma, and 200 mL of platelet concentrate were administered. After completion of operation, the patient was transferred to the intensive care unit with ventilator support under propofol sedation.

On postoperative day (POD) 2, the patient was weaned from mechanical ventilation. On POD 5, after our surgeons decreased the VAD dial from 3 to 2, the patient developed pulmonary congestion with respiratory distress, decreased arterial oxygen saturation, and elevated pulmonary artery pressure (PAP) (Table 2). Noninvasive positive-pressure ventilation (NPPV) was adopted, and intensified diuretic therapy was started. Additionally, the intensivists returned the VAD dial from 2 to 3. A few days later, he recovered from congestive heart failure, and then, NPPV was stopped. Following this, the patient did well hemodynamically and had good VAD performance. Another important point for postoperative management of VAD implantation is to prevent thrombosis. In this case, continuous infusion of heparin was administered for anticoagulation. The heparin infusion adjusted to an activated partial thromboplastin time (APTT) ration of 1.5–2.0 (or 40–50 s). Actually, the infusion rate or heparin was 15,000–20,000 units per day.

**Table 2:** Postoperative hemodynamic parameters and water balance

POD	0	1	2	3	4	5	6	7	8	9	10
CO (L/min)	4.8	5	5.3	5.1	5.2	5.1	5.5				
PAP (mmHg)	30/17/23	38/24/29	43/27/34	42/22/31	52/33/41	51/34/40	45/25/33				
CVP (mmHg)	10	12	12	12	13	13	13	14	12	13	11
Dial of Jarvik 2000*	3	3	3	3	3	2	3	3	3	3	3
Infusion volume (mL)	7984	3476	2937	2765	2806	3154	2968	2535	2758	2927	2928
Water output (mL)	3818	1297	1895	2055	2325	3245	2790	2760	2510	3940	2190
[Urine (mL)]	− 2864	− 787	− 1490	− 1815	− 2225	− 3195	− 2730	− 2660	− 2430	− 3870	− 2180
Water balance (mL)	4166	2179	1042	710	481	− 91	178	− 225	248	− 1013	738
Weight (kg)	60	62.1	62.6	63.5	63.7	64	62.5	62.9	62.2	60.6	59
Remarks		a				b				c	

*POD* postoperative day, *CO* cardiac output, *PAP* pulmonary artery pressure, *CVP* central venous pressure

^a^Weaning from ventilator and dobutamine. ^b^Emergence of pulmonary congestion and initiation of noninvasive positive-pressure ventilation and intensive diuretic therapy. ^c^Weaning from noninvasive positive-pressure ventilation

*The Jarvik 2000 pump speed and output were 10,000 rpm and 3–5 L/min on dial 3 and 9000 rpm and 2–4 L/min on dial 2

**Fig. 3:**
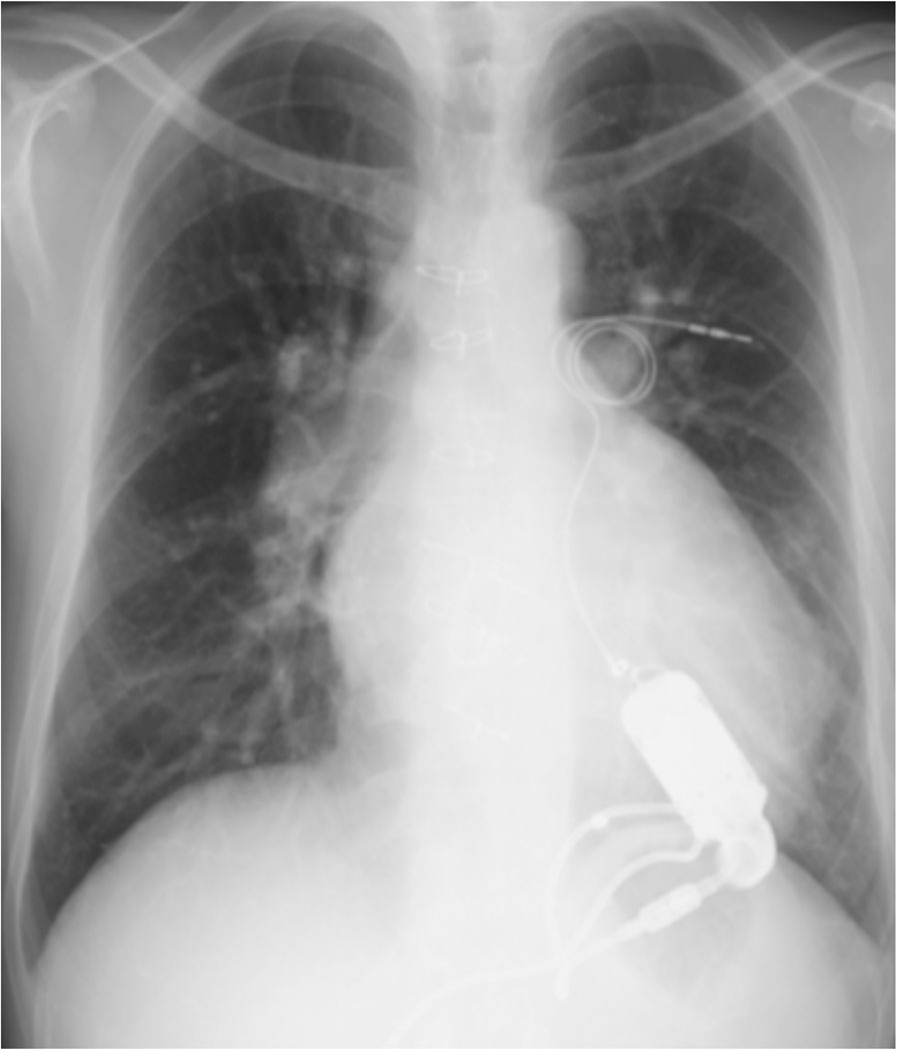
Chest radiography on postoperative day 90 shows a reduced ventricular shadow and the ventricular assist device (VAD)

The patient was discharged from the hospital on POD 60. He is now eagerly awaiting heart transplantation. Table 1 shows his hemodynamic data, and Fig. 3 shows his chest radiography on POD 90.

## Discussion

Many children with congenital heart disease (CHD) are now surviving into adulthood because of improved surgical repair techniques and management [1]; however, the number of adult congenital heart disease (ACHD) patients with heart failure is also increasing concomitantly [2].

Senning performed the first atrial switch procedure for TGA in 1958 [3], and Mustard developed an alternative operation, which resected the atrial septum and created an interatrial baffle to divert blood from both the superior vena cava and the inferior vena cava to the mitral valve and left ventricle [4]. Systemic RV dysfunction is a critical complication after a Senning or Mustard procedure because a morphological RV must support the systemic circulation instead of the morphological left ventricle [5]. Following an atrial switch operation, late death occurs in 16% of patients [6]. Additionally, the systemic RV is impaired in 9.1% of patients, and 52–55% of patients have a New York Heart Association functional class of II or III. Currently, the 20-year survival rate is 85.4% [7].

Implantation of VAD in a patient with ACHD, such as after an atrial switch operation for TGA, could be a particularly challenging procedure. A small number of cases describe the implantation of VAD for a morphologic RV [8–12]. Because the trabeculae carneae of the RV could disturb the blood flow to the inflow cannula, it must be resected via the RV apex before introduction of the inflow cannula to the apex of the RV, which was pointed out in this paper. Moreover, because cardiac surgery for ACHD involves the division of adhesions, the risk of bleeding, and challenging anatomy, VAD placement in ACHD patients is controversial [13].

The patient developed pulmonary congestion with increased PAP at POD 5. We presume that the preserved pulmonary LV function and decreased VAD output could easily induce over-filling of the systemic RV in patients after an atrial switch operation. In a previous report, Toyama et al. suggest that it is essential to adjust the systemic VAD flow according to the pulmonary ventricular function and the systemic ventricular filling in case of VAD implantation to a patient with CCTGA [14].

In fact, this case had congestive heart failure following the decrease in VAD dial by the surgeons from 3 to 2 on POD5. Our surgeons decreased the VAD dial in order to open aortic valve because they feared thrombosis at the aortic root. We thought that this procedure could induce pulmonary congestion. Actually, after the intensivists returned the VAD dial from 2 to 3, he recovered from congestive heart failure in combination with NPPV adaptation and the intensified diuretic therapy.

Although he underwent surgical repair for stenosis of his pulmonary veins at 2 years of age, there was the potential for residual pulmonary vein obstruction (PVO). In this case, PVO could be one of the major risk factors for pulmonary congestion. However, we unfortunately did not examine for details in this case, and so we could not deny or affirm the existence of PVO. In the perioperative management of VAD implantation for ACHD patients, we must assess the presence or absence of PVO preoperatively. PVO is a congenital or acquired cardiac malformation that is hard to diagnose clinically and could be properly diagnosed by echocardiography, and cardiac catheterization could reveal the site of stenosis morphologically. Additionally, because the volume of the atrium was limited after atrial baffle repair, excessive transfusion could easily induce pulmonary congestion.

Postoperative anticoagulant therapy of patients undergoing VAD implantation is one of the most important issues. Anticoagulation protocols change by each institution, implanted device, and individual patient. In many cases, to prevent clot formation, both warfarin and antiplatelet drugs, such as aspirin or dipyridamole, are administered for anticoagulation [15]. During warfarin therapy, the target prothrombin-international normalized ratio (PT-INR) ranges from 2.5 to 3.0. Aspirin is often given in doses from 80 to 325 mg per day*.* During anticoagulant therapy, we must pay attention to systemic bleeding complications.

VAD placement for systemic RV failure is a life-saving treatment in ACHD patients who encounter end-stage heart failure. Although these patients have unique anatomical and physiological characteristics that can make clinical management more challenging, systemic VAD implantations with systemic RV dysfunction have the potential to improve physiological conditions and can offer a bridge to heart transplantation.

## Abbreviations

ACHD: Adult congenital heart disease
CCTGA: Congenitally corrected transposition of the great arteries
CHD: Congenital heart disease
CPB: Cardiopulmonary bypass
LV: Left ventricular
NPPV: Noninvasive positive-pressure ventilation
PAP: Pulmonary arterial pressure
POD: Postoperative day
RV: Right ventricular
TGA: Transposition of the great arteries
VAD: Ventricular assist device

## Authors’ information

KS is a M.D. and staff anesthesiologists of the Anesthesiology and Perioperative Medicine, Tohoku University School of Medicine. HT is a M.D., Ph.D., and lecturer of the Department of Anesthesiology, Tohoku University Hospital. NA is a M.D. and staff anesthesiologists of the Department of Anesthesiology, Tohoku University Hospital. AS is a M.D. and clinical fellow of the Department of Anesthesiology, Tohoku University Hospital. YE is a M.D., Ph.D., and Professor of Surgical Center and Supply, Sterilization, Tohoku University Hospital. MY is a M.D., Ph.D., and Professor of Anesthesiology and Perioperative Medicine, Tohoku University School of Medicine.
